# Method for Simulating the Anti-Damage Performance of Consolidation Soil Balls at the Roots of Seedlings during Transportation Using Consolidated Soil Columns

**DOI:** 10.3390/polym15204083

**Published:** 2023-10-14

**Authors:** Shaoli Wang, Shengju Song, Xuping Yang, Zhengqi Xiong, Chaoxing Luo, Donglu Wei, Hong Wang, Lili Liu, Xinxin Yang, Shaofeng Li, Yongxiu Xia

**Affiliations:** 1 State Key Laboratory of Tree Genetics and Breeding, Experimental Center of Forestry in North China, National Permanent Scientific Research Base for Warm Temperate Zone Forestry of Jiulong Mountain in Beijing, Chinese Academy of Forestry, Beijing 100091, China; wshaoli@iccas.ac.cn (S.W.); lisf@caf.ac.cn (S.L.); 2 R&D Center, China Academy of Launch Vehicle Technology, Beijing 100076, China; songshengju99@163.com; 3 Security Department, Chinese Academy of Forestry, Beijing 100091, China; yxp@caf.ac.cn; 4 College of Material and Chemical Engineering, Heilongjiang Institute of Technology, Harbin 150050, China; xiongzhengqi1020@163.com (Z.X.); luochaoxing525@163.com (C.L.); mit_ing@163.com (D.W.); wanghongkey@126.com (H.W.); liulili760802@126.com (L.L.); 5 Management Center of Songshushan Nature Reserve, Inner Mongolia, Songshushan Forestry Center, Wengniute Banner, Chifeng 024500, China; yangxinxin717@163.com

**Keywords:** anti-transport vibration, compressive strength, consolidated soil column, damage resistance, polymer-based consolidation agents

## Abstract

In the process of landscaping or afforestation in challenging terrain, in order to improve the survival rate of transplanted seedlings, it is necessary to transplant seedlings with a mother soil ball attached. During transportation, the soil ball at the root of the seedlings is very susceptible to breakage due to compression, bumps, and collisions. In order to ensure the integrity of the soil ball of the transplanted seedlings and improve the survival rate of seedlings, a method of chemically enhancing the soil surface strength was employed. Specifically, a polymer-based soil consolidating agent was used to solidify the root balls of the seedlings. To examine the abrasion resistance performance of the soil balls formed by consolidating the surface with polymer adhesive during the transportation process, we utilized a polymer-based consolidating agent to prepare test soil columns and developed a method to simulate the damage resistance performance of seedling root balls during transportation using these soil columns. The method primarily encompasses two aspects of testing: compressive strength testing of the consolidated soil columns and resistance to transportation vibration testing. The first method for testing the resistance to transportation vibration of the consolidated soil columns is a combination test that includes three sets of tests: highway truck transportation vibration testing, combined wheel vehicle transportation vibration testing, and impact testing. Although the method is cumbersome, testing is more accurate. The second method for testing the resistance to transportation vibration of the consolidated soil columns involves simultaneously testing multiple consolidated soil columns using a simulated transportation vibration test platform. The testing method is concise and efficient, and the test results are more intuitive. The combined assessment of the resistance to transportation vibration and compressive strength testing of the consolidated soil columns allows for a comprehensive evaluation of the soil columns’ resistance to damage during transportation. This study mainly provides a quick and effective method for detecting the damage resistance of consolidated soil columns/balls during transportation, providing technical support for the application of polymer-based consolidation agents in the field of seedling transplantation.

## 1. Introduction

In order to maintain a good ecological environment and urban living environment, it is necessary to strengthen afforestation and urban greening [1,2,3,4,5,6]. Whether it is afforestation or urban greening, a large number of seedlings need to be transplanted [7,8]. The most commonly employed method is to guarantee the intactness of the root ball of the transplanted seedlings to ensure the survival rate of transplanted seedlings [9,10]. The diameter and height of the soil ball at the root of the transplanted seedlings are proportional to the diameter at breast height (DBH) of the transplanted seedlings [11]. Generally, the thicker the DBH of transplanted seedlings, the larger the soil ball requirement at the root of the seedlings [12,13,14]. Due to the loose soil in the nursery, the soil ball has a low degree of consolidation. After successfully raising seedlings from the nursery, the soil balls at the roots of the seedlings are easily broken during transportation due to squeezing, bumping, and mutual impact [13,14,15]. In order to ensure the integrity of the soil ball at the root of seedlings, commonly employed methods currently include wrapping, binding, or wooden crate packaging, among others. However, these methods present two primary issues: (1) Some of the materials used for wrapping and binding, such as plastic ropes, plastic sheets, and iron wires, are not readily biodegradable, thus posing a risk of environmental pollution; (2) the process of wrapping, binding, and lifting soil balls places high technical demands on workers. Consequently, the lifting process not only consumes a substantial amount of labor and resources but also results in low efficiency. The key is that it is difficult to ensure the integrity of soil balls in the end, especially during the loading, unloading, and transportation of seedlings, where the mother soil ball is often damaged. Among the causes of death of transplanted seedlings, soil ball breakage is the main cause [8,12,13,16].

Solidifying agents that have good consolidation properties for soil cannot be directly used to consolidate soil balls at the roots of seedlings. As is well known, the soil treated with cement/fly ash is not only brittle and prone to cracking, but the key is that the formed consolidated surface cannot revert from its consolidated state to loose soil. Inorganic solidifying agents can only solidify the soil and cannot depolymerize, belonging to a single type of solidifying agent [17]. The key is that these solidifying agents can also inhibit plant growth and have negative effects on the environment [18,19,20,21]. In the field of geotechnical engineering, the utilization of biopolymers has many advantages. When biopolymers are mixed with soil, they can cause changes in soil properties, such as increased compressive strength, improved erosion resistance, decreased permeability, and suitability for vegetation growth [22,23,24]. Compared with other biological methods, direct use of biopolymers also has the advantages of shorter processing time, no need for microbial or nutrient injection, and compatibility with clay soils. Due to the natural existence of biopolymers in nature, and many of them being harmless and even edible, biopolymers are considered a sustainable and environmentally friendly building material. As a binder, biopolymers can not only directly improve the combination between soil particles but also form gel-like films around soil particles [22]. Therefore, choosing biodegradable polymer-based soil consolidation agents is the optimal choice for consolidating the root ball of seedlings.

Based on this, in response to the demand for the integrity of the mother soil ball during seedling transplantation, our research team prepared environmentally friendly and degradable polymer-based soil consolidation agents using biodegradable and environmentally friendly materials such as konjac glucomannan (KGM) [25,26,27,28,29,30], chitosan (CA) [31,32,33,34], and polyvinyl alcohol (PVA) [35,36] as the main raw materials in the previous research work [13,14,15,16]. The prepared KGM/CA/PVA soil consolidation agent was uniformly sprayed to the surface of the soil ball at the root of the transplanted seedlings. Once this consolidating agent had dried, it formed a rigid, net-like film together with the soil on the surface of the root ball. We have observed that after completing the transplanting task, the KGM/CA/PVA net-like gel film formed on the surface of the root ball of transplanted seedlings gradually degraded [13,14,15]. Furthermore, the degradation products were non-polluting to groundwater and soil, and they did not adversely affect the growth of the seedlings [13,14,15,20]. Most importantly, the KGM/CA/PVA net-like gel film formed on the surface of the root ball provided protection to the transplanted seedlings’ root balls, reducing wear and tear during transportation. This film enhanced their resistance to transportation-induced vibrations and compression. Hence, it is evident that using biodegradable polymer-based consolidation agents can effectively stabilize the root ball of seedlings. However, it should be noted that the preparation conditions of the KGM/CA/PVA consolidation agent, such as the preparation temperature of the solution, the solid content of KGM, CA, and PVA in the solution, and the pH of the solution, all have an impact on various properties of the consolidation agent. These factors can influence the viscosity, flowability, moisture permeability, breathability, and the consolidating performance of the film on soil columns, among other characteristics. Indeed, the consolidation effectiveness of KGM/CA/PVA consolidation agents on soil can vary under different preparation conditions. However, during the process of transplanting seedlings, it is required that the consolidated soil ball should be able to withstand the vibrations and friction during the transportation of the transplanted seedlings from the nursery to the planting location. Therefore, it is imperative to conduct testing experiments to assess the consolidation effectiveness of soil balls treated with the KGM/CA/PVA consolidation agent in response to the various challenges encountered during the transportation of transplanted seedlings on highways or national roads, including oscillations and friction, sudden drops, impacts caused by road irregularities, and mutual squeezing between soil balls during whole-vehicle transportation with soil-encased seedlings [13,14,15].

Through our research, it has been observed that the primary method for evaluating the soil consolidation effectiveness of polymer-based consolidation agents is the use of static compaction tests. In this method, test specimens are prepared according to ASTM standards D2266-23 [37] and D3080-98 [38]. Unconfined compression specimens are created with dimensions of 8 cm in height and 3.91 cm in diameter, while direct shear specimens have dimensions of 2 cm in height and 6.18 cm in diameter. Then, the prepared sample is consolidated at 25 °C for 48 h and then subjected to unconfined compressive strength and shear strength tests. These types of tests primarily characterize the consolidation effects of soil on the surface of road slopes, and they are considered static tests. Indeed, during the transportation of transplanted seedlings with soil-encased root balls, the phenomena involving friction and squeezing between soil balls are dynamic processes. Consequently, it is necessary to explore new testing methods to assess the consolidation performance of polymer-based consolidation agents on the root balls of seedlings in such dynamic scenarios [13,14,15].

In light of this, our research team employs two characterization methods, namely the compression strength test of consolidated soil columns and the vibration resistance test during transportation, to comprehensively evaluate the consolidation effectiveness of polymer-based soil consolidation agents on soil columns. A test soil column was prepared using a KGM/CA/PVA consolidation agent, and the compressive strength of different consolidated soil columns was tested using a universal testing machine. The influence of preparation conditions of consolidated soil columns on their compressive strength was explored. Then, two different anti-transport vibration properties were used to characterize the influence of preparation conditions of consolidated soil columns on their anti-transport performance. This work primarily presents a novel, efficient method for rapidly assessing the dynamic damage resistance of consolidated soil columns/balls during transportation. Through this innovative, dynamic damage resistance testing method, it becomes possible to evaluate how the preparation conditions of polymer-based soil consolidation agents and soil columns impact the damage resistance performance of the consolidated soil columns. This methodology enables the identification of optimal conditions for both the consolidation agent and soil columns, laying the foundation for the widespread application of KGM/CA/PVA polymer-based soil consolidation agents in forestry.

## 2. Materials and Methods

### 2.1. Experimental Materials and Equipment

(1) Experimental materials

Konjac flour (KGM, 200 g/bottle), Chitosan (CA, chemical pure), polyvinyl alcohol (PVA, superior-grade pure), sodium hydroxide (analytical purity), acetic acid (excellent-grade pure), and other compounds were supplied by Sinopharm Chemical Reagent Co., Ltd. of China, Shanghai, China.

(2) Experimental equipment

Electronic Analytical Balance; pH meter; thermostat water bath; mechanical stirrer; DV3T Viscometer; Universal testing machine (LGD3000); Simulated transportation vibration test bench MPA3324/H1248A (10T) and LS437A/BT900M (4T); Simulated transportation vibration test bench HK-120 with a payload of 300 kg.

### 2.2. Experimental Methods

(1) Preparation of polymer-based soil consolidation agent

**Figure 1:**
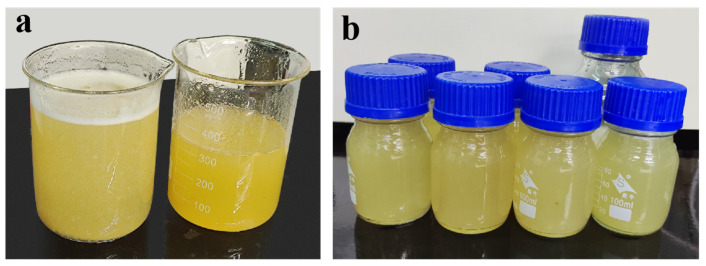
KGM/CA/PVA ternary blend adhesive. (**a**) Main adhesive of prepared KGM/CA/PVA polymer adhesive; (**b**) KGM/CA/PVA soil consolidation agent with additives added.

Similar to the previous preparation method [13,14,15,16], first prepare a polyvinyl alcohol solution with a mass fraction of 10%. Then, in a 20% concentration of a glacial acetic acid solution, adjusting the preparation temperature, pH, and the ratio of KGM and CA, the KGM/CA binary blend adhesive can be obtained by mechanical stirring in a four-necked flask and using polymer blending preparation technology. Finally, add 10% PVA to the mixed solution and stir for 1 h to prepare the KGM/CA/PVA ternary blend solution, as shown in Figure 1.

(2) Preparation of soil column samples

As the previous preparation method [13,14,15], firstly, dry the soil thoroughly in the oven. Weigh 400 g of dried soil, mix it with 100 g of pure water, and stir evenly. The molds for soil columns can be categorized into two distinct types: Type 1 molds possess a diameter of 50 mm and a height of 50 mm, while Type 2 molds feature a diameter of 100 mm and a height of 64 mm. When utilizing Type 1 molds for the preparation of soil columns (Model 1), approximately 100 g of moist soil is required. In contrast, the use of Type 2 molds for crafting soil columns (Model 2) necessitates approximately 400 g of moist soil. After removing the mold, the KGM/CA/PVA blending adhesive needs to be coated to the surface of the soil columns. The consolidated soil column can be obtained by drying and consolidating at room temperature for 24 h. The amount of adhesive used on the surface of the same type of soil column is controlled equally: Model 1 requires approximately 10 g of adhesive, while Model 2 requires approximately 40 g of adhesive.

(3) Compressibility test of consolidated soil column

Use an INSTRON 5582 universal testing machine to test the compressive strength of consolidated soil columns. The time interval for sample testing is 0.5 s, and the compression rate is 1 mm/min [13,14].

(4) Test methods for transportation vibration resistance of consolidated soil columns

**Figure 2:**
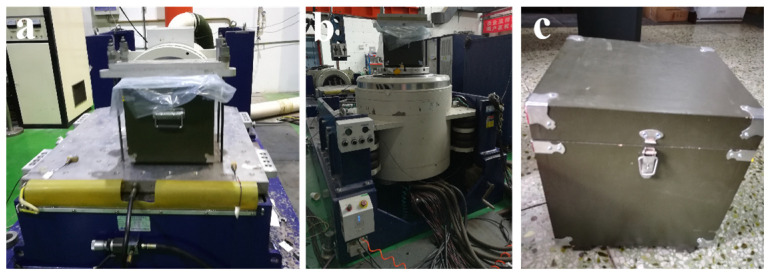
Vibration resistance testing of consolidated soil columns. (**a**) LS437A/BT900M(4T) Vibration Table; (**b**) MPA3324/H1248A (10T) vibration table; (**c**) Sample box.

Test Method 1: According to Part 16 “Vibration Test” and Part 18 “Impact Test” of GJB150.16A-2009 “Environmental Testing Methods for Military Equipment Laboratories-Vibration Test” [39], the simulated transportation anti-vibration performance of consolidated soil columns is tested. The testing content includes highway truck vibration test, combined wheel vehicle vibration test, and impact test. Horizontal, longitudinal, and vertical tests were conducted on highway truck transportation oscillation tests, combined wheel vehicle transportation vibration tests, and impact tests. The lateral and longitudinal tests of highway truck vibration test, combined wheel vehicle vibration test, and impact test are all completed by the LS437A/BT900M (4T) vibration table, while the vertical tests of highway truck vibration test, combined wheel vehicle vibration test, and impact test are all completed by the MPA3324/H1248A (10T) vibration table, as shown in Figure 2 [14,15].

The test conditions for the highway truck vibration test are shown in Table 1. Under the test conditions, the transportation mileage of the simulated highway reached over 1000 km.

The test conditions for the vibration test of combined wheeled vehicles are shown in Table 2. The vibration test of combined wheeled vehicles simulates the vibration situation during transportation on national highways of third and fourth levels. According to the test conditions in Table 2, the transportation mileage of the simulated Class III and IV national roads reached over 400 km.

**Figure 3:**
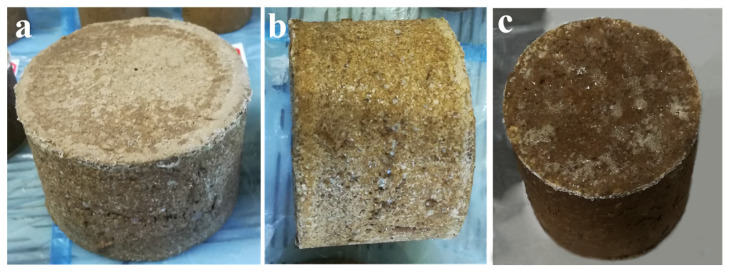
Prepared consolidated soil column. (**a**) Front of consolidated soil column model 2; (**b**) Sides of consolidated soil column model 2; (**c**) Front of consolidated soil column model 1.

The test conditions for the impact test are shown in Table 3. After the consolidated soil column samples are packed in sealed bags, they are closely arranged in the test box in order, and the gaps in the box are filled with plastic foam, as shown in Figure 3a–c. Then, fix the box on the corresponding vibration table according to the testing process and start the corresponding testing program according to the testing requirements to begin testing.

**Figure 4:**
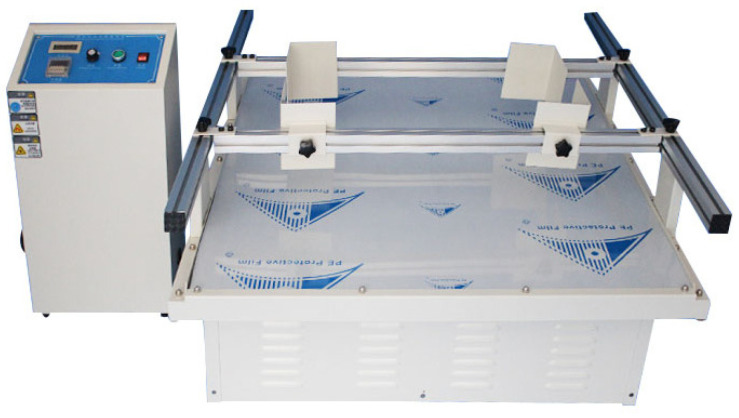
Vibration resistance testing of consolidated soil columns.

Test Method 2: According to the testing standards of the American Society for Transportation (ISTA) and the American Society for Materials (ASTM), as shown in Table 4, the transport vibration resistance of consolidated soil columns was tested using a simulated transport vibration test bench HK-120 (Figure 4). The size of the box used for testing is 33 cm × 22 cm × 24 cm. Arrange two layers of consolidated soil columns neatly inside the box, number them and place them randomly, without any protective measures against wear and tear, to ensure that each consolidated soil column is evenly stressed during oscillation. The specific testing indicators are shown in Table 4 [13,15].

## 3. Results

### 3.1. Compressive Performance of Consolidated Soil Column

During the transportation of soil balls, the mutual compression between soil balls and the impact resulting from the jolts during transit can lead to significant damage to the integrity of tree seedling root balls. After solidifying the surface of the soil column with the polymer-based adhesive film, the consolidation adhesive film can provide effective protection for the integrity of the soil column.

**Figure 5:**
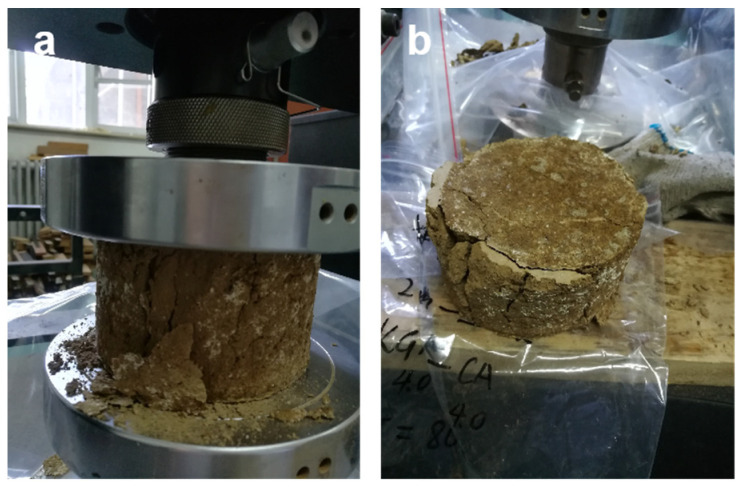
Test method for compressive strength of consolidated soil columns. (**a**) Compressive strength testing of consolidated soil columns; (**b**) Consolidated soil column after compression.

We utilized a universal testing machine to test the compressive strength of consolidated soil columns to evaluate their compressive performance during transportation, as shown in Figure 5. Previous research has indicated that the preparation conditions of polymer-based soil consolidating agents, such as preparation temperature, pH of the adhesive solution, solid content of the adhesive solution, as well as soil particle size and pH, can indeed have a significant impact on the compressive strength performance of the consolidated soil column [13,14].

In the earlier stages of research, it was observed that with the elevation of the adhesive solution temperature, an increase in pH, and higher concentrations of KGM, CA, and PVA, there was an overall enhancement in the compressive strength of the consolidated soil column. Furthermore, it was also observed that as the soil particle size increased, the compressive strength of the consolidated soil column increased. Additionally, as the soil pH increased, the compressive strength of the consolidated soil balls initially increased and then reached a stable state [13,14,15].

Based on the previous research results [13,14,15], two types of soil columns were prepared using the same polymer-based adhesive, as shown in Table 5. The primary focus of this study was to investigate the influence of variations in soil particle size and mixed particle size (undisturbed soil) on the consolidation behavior of these soil columns. Simultaneously, the study examined the effects of the size and dryness of consolidated soil columns on their compressive strength.

**Figure 6:**
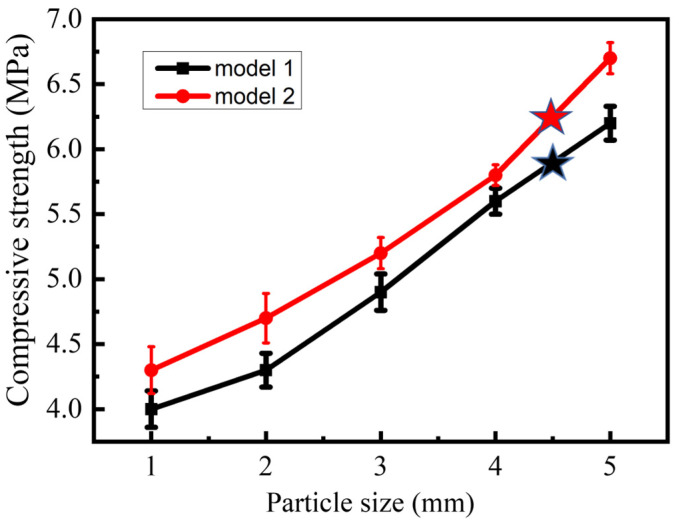
The effect of soil particle size on compressive strength of consolidated soil columns. The black star represents the compressive strength of sample 6; The red star represents the compressive strength of sample 12.

From Figure 6, it can be seen that as the soil particle size increases, the compressive strength of the consolidated soil column gradually increases. Samples 6 and 12 of consolidated soil columns were prepared directly from the soil with mixed particle sizes, aiming to simulate the authentic structure of soil balls around tree seedling roots. During the research process, it was found that the compressive strength of the consolidated soil column was between that of the consolidated soil column with a soil particle size between 4 mm and 5 mm, as shown in Figure 6. It was found that when the soil particle size is the same, the larger the consolidated soil column, the greater its compressive strength.

**Figure 7:**
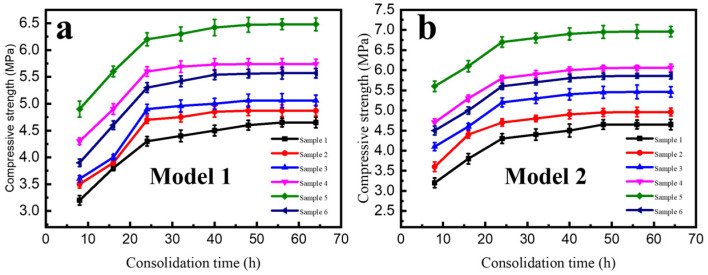
The effect of consolidation time of adhesive on the compressive strength of consolidated soil columns. (**a**) Effect of different consolidation times on consolidation strength in Model 1 of consolidated soil column; (**b**) Effect of different consolidation times on consolidation strength in Model 2 of consolidated soil column.

Following the application of the KGM/CA/PVA polymer-based soil consolidation agent onto the surface of the soil columns, the current adhesive undergoes a drying period of 8–24 h on the soil column surface, resulting in the surface hardening of the consolidated soil columns. In our research, we observed that in two different types of consolidated soil columns (Model 1 and Model 2), as the consolidation time extended from 8 h to 24 h, the compressive strength of the consolidated soil columns increased slightly faster. However, when the consolidation time was further extended from 24 h to 48 h, the rate of increase in compressive strength of the consolidated soil columns slowed down. After a consolidation time exceeding 48 h, the compressive strength of the consolidated soil columns remained relatively constant, as illustrated in Figure 7a,b. Overall, in Model 2, the compressive strength of the consolidated soil column is slightly higher than that in Model 1, as shown in Figure 7a,b.

### 3.2. The Main Influencing Factors of Transportation Oscillation Resistance

#### 3.2.1. Main Influencing Factors on the Transportation Oscillation Resistance of Consolidated Soil Columns in Test Method 1

(1) The influence of transportation-induced vibration modes on the consolidated soil columns

Firstly, we conducted tests on the resistance of the consolidated soil columns to transportation-induced vibrations using Method 1. The research findings indicate that, under the testing conditions outlined in Table 1 and Table 3, no wear or breakage was observed in the consolidated soil columns during the vibration tests associated with highway truck transportation and impact tests in three directions.

Under the testing conditions specified in Table 2, during the lateral and longitudinal tests of the combined-wheel vehicle transportation-induced vibration, no wear or breakage of the consolidated soil columns was observed. However, in the vertical testing process, varying degrees of wear and fracture were noticed in the tested consolidated soil columns. It was found that factors such as the type of consolidation adhesive film, the testing location of the consolidated soil columns, and the moisture content of the consolidated soil columns all have an impact on their wear resistance performance.

(2) The influence of the flowability of the consolidation adhesive on the resistance of consolidated soil columns to transportation-induced vibrations

**Figure 8:**
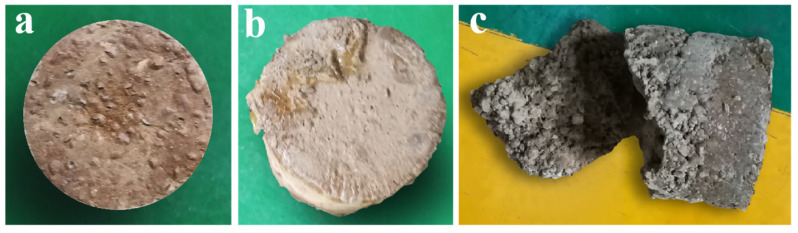
The influence of consolidation agent types on resistance of consolidated soil columns to transportation-induced vibrations. (**a**) The state of the consolidated soil column prepared under the preparation conditions of Type 1 after the oscillation test of Method 1; (**b**) The state of the consolidated soil column prepared under the preparation conditions of Type 2 after the oscillation test of Method 1; (**c**) The state of the consolidated soil column prepared under the preparation conditions of Type 3 after the oscillation test of Method 1.

Consolidated soil columns were prepared by applying the first two types of KGM/CA/PVA polymer-based soil consolidation agents in Table 6, which exhibit better flowability, onto the surface of the soil columns. During the testing process, this type of consolidated soil column was placed in the center position of the testing box. It was found that this specific type of consolidated soil column demonstrated excellent resistance to vibrations, with no observable wear, as illustrated in Figure 8a,b.

The third type of KGM/CA/PVA polymer-based soil consolidation agent from Table 6 exhibited poor flowability. Even when using larger soil particles to prepare consolidated soil columns, the adhesive film is not only difficult to penetrate into the gaps inside the soil column due to its viscosity but also difficult to form a uniform adhesive film on the surface of the soil column. As a result, the adhesion of the consolidation film to both the surface and interior of the soil columns was comparatively weak. During the vibration testing process, it was difficult to maintain the integrity of the consolidated soil columns, as shown in Figure 8c.

(3) The influence of the drying degree of the consolidation film on its resistance to transportation-induced vibrations

**Figure 9:**
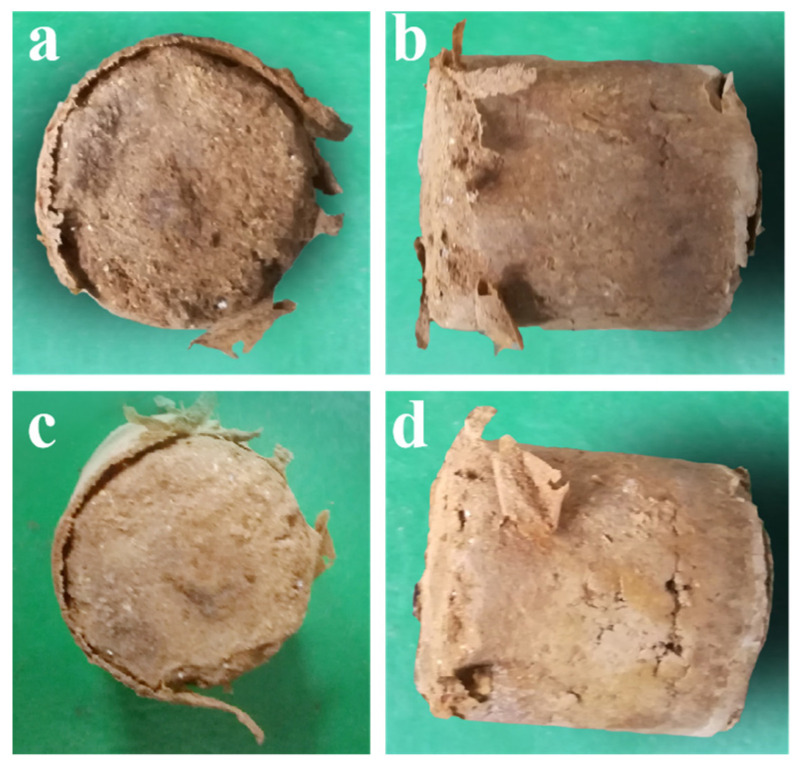
Consolidated soil column with surface wear. (**a**) The upper surface wear of the consolidated soil column with a drying time of 24 h after the oscillation test of Method 1; (**b**) The side surface wear of the consolidated soil column with a drying time of 24 h after the oscillation test of Method 1; (**c**) The upper surface wear of the consolidated soil column with a drying time of 30 h after the oscillation test of Method 1; (**d**) The side surface wear of the consolidated soil column with a drying time of 30 h after the oscillation test of Method 1.

During the testing process, it was found that there was significant wear on the contact surface between the two layers of consolidated soil columns in the testing box, especially on the upper and lower surfaces of the consolidated soil columns, as shown in Figure 9. This suggests that the consolidation film plays an essential role in protecting the surface of the consolidated soil columns during transportation-induced vibrations.

**Figure 10:**
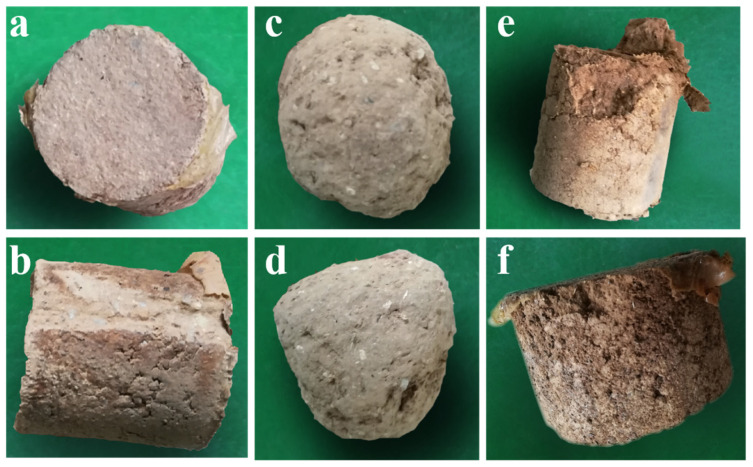
Worn and fragmented consolidated soil columns. (**a**) The upper surface wear of the consolidated soil column with a drying time of 50 h after the oscillation test of Method 1; (**b**) The side surface wear of the consolidated soil column with a drying time of 50 h after the oscillation test of Method 1; (**c**) After the surface consolidation adhesive film of the consolidated soil column in (**a**) was worn, the state of the upper surface of the soil column after oscillation testing using Method 1 again; (**d**) After the surface consolidation adhesive film of the consolidated soil column in (**a**) was worn, the state of the side surface of the soil column after oscillation testing using Method 1 again; (**e**) After the oscillation test of Method 1, the surface fragmentation state of Model 1’s consolidated soil column with a drying time of 18 h; (**f**) After the oscillation test of Method 1, the surface fragmentation state of Model 2’s consolidated soil column with a drying time of 18 h.

From Figure 9 and Figure 10a,b, it is evident that consolidated soil columns with longer drying times exhibit superior resistance to transportation-induced vibrations and are less prone to fracture. In the testing location at the side of the testing box, during the transportation-induced vibration tests, the side surfaces of the consolidated soil columns are susceptible to abrasion by the testing box itself, as shown in Figure 10b.

As the vibration frequency increases, after the consolidation film has been completely worn away, the soil column’s wear resistance performance notably decreases, as illustrated in Figure 10c,d. From Figure 10e,f, it can be observed that consolidated soil columns with shorter drying times display slightly lower resistance to transportation-induced vibrations, with the edges of the consolidated soil columns being more susceptible to breakage during the vibration tests.

#### 3.2.2. Main Influencing Factors on the Transport Vibration Resistance of Consolidated Soil Columns in Test Method 2

**Figure 11:**
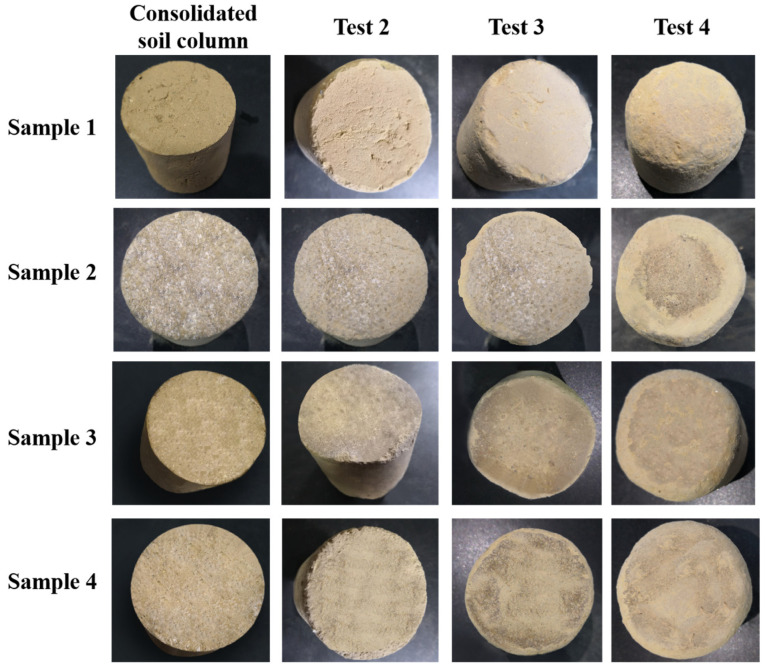
Wear state of consolidated soil columns tested using Method 2.

According to the testing method in Table 4, we conducted tests on the resistance to transportation-induced vibrations of four consolidated soil column samples listed in Table 7 using the simulated transportation vibration platform HK-120 (as shown in Figure 4). After conducting Test 1, it was observed that the consolidated soil columns had almost no wear, so the image was not shown in Figure 11.

As the vibration frequency gradually increased, the wear on the four consolidated soil column samples increased progressively. Compared with the other samples, control sample 1, which was not coated with the consolidation film, exhibited the most severe wear. This suggested that the polymer-based KGM/CA/PVA consolidation film provided a certain level of protection for the soil columns. The other three samples sprayed with consolidation adhesive showed relatively low wear in the vibration tests of Test 2 and Test 3. In Test 4, it was found that the wear of samples 3 and 4 was smaller than that of sample 2. From this, it can be seen that the higher the preparation temperature of the adhesive, the better the wear resistance of the prepared consolidated soil column.

According to the previous research results [13,14,15], the testing frequency of Test 4 is closest to the actual test conditions for resistance to transportation oscillations. Therefore, the test results of Test 4 have real reference values for consolidated soil columns in real transportation environments.

## 4. Discussion

### 4.1. Main Factors Affecting Compressibility of Consolidated Soil Column

#### 4.1.1. Analysis of the Influence of Soil Particle Size on the Compressive Resistance of Consolidated Soil Columns

The compressive strength tests on the consolidated soil columns primarily simulate the compressive performance between consolidated soil balls under conditions similar to when tree seedlings with soil balls are transported and encounter rough terrain. During this process, the soil balls around the tree roots may get jostled and collide with each other as the transport vehicle encounters uneven terrain, resulting in compression forces between them.

From previous research, it can be observed that the preparation conditions of the consolidation agent impact its viscosity and flowability. A higher viscosity of the adhesive led to better adhesion to the consolidated soil columns. In cases where the soil particle size is the same, there is better flowability of the adhesive, which results in relatively better penetration of the adhesive into the soil. When preparing consolidated soil columns, this resulted in a thicker consolidation film formed by the adhesive in conjunction with the soil.

Indeed, as shown in Figure 6, it becomes evident that larger soil particle sizes result in better permeability of the adhesive, leading to a thicker consolidation film formed by the adhesive and soil on the surface of the consolidated soil columns. Consequently, these consolidated soil columns exhibited improved resistance to compression. Additionally, as observed in Figure 6 for samples 6 and 12, when using soil with non-uniform particle sizes to prepare consolidated soil columns, the uneven voids within the soil column led to non-uniform consolidation film thickness, which can also affect the corresponding consolidated soil columns’ compressive performance.

#### 4.1.2. Analysis of the Influence of Drying Degree of Adhesive Film on the Compressive Resistance of Consolidated Soil Columns

After the adhesive was sprayed onto the surface of the soil columns, it dried relatively quickly due to direct exposure to the air, forming a hardened film on the surface within 8 h, as observed in the appearance of the soil columns. However, within the interior of the consolidated soil columns, the drying process of the adhesive was slower. As depicted in Figure 7, the adhesive inside the consolidated soil columns was mostly dried by 24 h and completely dried by 48 h. A thoroughly dried consolidation film offered better protection for the consolidated soil columns.

### 4.2. Main Factors Affecting the Transport Vibration Resistance of Consolidated Soil Columns

#### 4.2.1. Analysis of the Influence of Adhesive Film on the Transport Vibration Resistance of Consolidated Soil Columns

Through the anti-transportation vibration test conducted on the soil column using Method 1, it can be found that the consolidation adhesive film on the surface of the soil column has a good protective effect on the consolidation soil column/soil ball during highway transportation. The consolidation adhesive film formed by selecting KGM/CA/PVA adhesive with moderate viscosity and fluidity can effectively protect the integrity of the consolidated soil column/ball. Through the simulation test of Method 1, it can be seen that during the transportation process on third- and fourth-level national highways, the wear of the consolidated soil column was relatively small under the protection of the consolidated adhesive film.

At the same time, through the Test Method 2, it also found that the consolidation adhesive film had a good protective effect on the consolidation soil column. The preparation conditions of the consolidation adhesive have a significant impact on the quality of the consolidation adhesive film, and the degree of consolidation of the adhesive film will determine whether the integrity of the consolidation soil column can be ensured during the testing process.

From this, it can be seen that the presence of the adhesive film on the surface of soil columns significantly impacts the ability of consolidated soil columns to resist transportation-induced vibrations. The adhesive film serves as a protective layer, offering several key benefits:

(1) Enhanced structural integrity

The adhesive film adheres to the surface of the soil columns, providing increased cohesion and stability to the overall structure. This improved integrity helps the soil columns withstand external compression forces during transportation.

(2) Reduced wear and friction

The film reduces wear and friction between soil particles within the columns. This minimizes abrasion and collision between soil particles, which is common during transportation on uneven terrain.

(3) Uniformity of protection

Proper application of the adhesive film ensures uniform coverage on the soil column’s surface. This uniformity is crucial for consistent protection against oscillations and compression.

(4) Moisture retention

Depending on its properties, the adhesive film may help retain moisture within the soil columns. Adequate moisture content can contribute to the overall strength and resilience of the columns.

(5) Drying time

The drying time of the adhesive film is an important consideration. A well-dried film provides better protection, as it forms a robust and consistent layer. Longer drying times generally lead to more effective protection.

In conclusion, the adhesive film plays a pivotal role in enhancing the transportation vibration resistance of consolidated soil columns. It promotes structural integrity, minimizes wear, and ensures uniform protection, ultimately contributing to the columns’ ability to withstand the challenges of transportation-induced vibrations.

#### 4.2.2. The Influence of the Testing Position of Consolidated Soil Columns on Their Resistance to Transportation Vibration

During the testing process in Method 1, due to the close arrangement of consolidated soil columns, when simulating transportation-induced vibrations on third- and fourth-level national highways, there are simultaneous vibrations occurring in three different directions with varying frequencies. Due to the differences in vibration frequency and amplitude, the wear of consolidated soil columns at different positions in the test box varies. The consolidated soil columns positioned at the edges of the test box experience more friction with the box’s walls, leading to significant wear on the sides of these soil columns. In the central positions of the test box, during the initial stages of vibration, the closely arranged consolidated soil columns primarily experience wear concentrated between the upper and lower surfaces of the two layers of consolidated soil columns and between the bottom layer of consolidated soil columns and the bottom of the box. As the vibration testing progresses, there is also mutual wear, collision, and compression between the consolidated soil columns, leading to damage at the edges of the columns. If the consolidation film on the surface of the columns is relatively thick or if the overall drying degree of the consolidated soil columns is deep, the consolidation film provides effective protection, allowing the columns to maintain their integrity. If the consolidation film on the surface of the consolidated soil columns is relatively thin, with an increase in vibration frequency and amplitude, the film on the column’s surface can be completely worn away. Without the protective function of the consolidation film, the wear on the soil columns can become severe, as shown in Figure 10c,d.

From this, it can be seen that the position at which consolidated soil columns are tested can have a significant influence on their resistance to transportation-induced vibrations. Here is an analysis of this influence:

(1) Surface wear and impact

The testing position, whether on the surface or sides of the soil columns, affects the type and degree of wear and impact they experience during vibration tests. Soil columns tested on their sides may be more susceptible to abrasion and impact on their lateral surfaces due to contact with the testing equipment or adjacent columns.

(2) Uniformity of stress distribution

The position of testing can impact the uniformity of stress distribution within the soil columns. Soil columns tested on their sides may experience uneven stress patterns compared to those tested on their surfaces, potentially leading to differences in wear and deformation.

(3) Effect on consolidation film

The testing position can also affect the integrity of the consolidation film on the soil columns. When tested on their sides, there may be differential pressure and shear forces acting on the consolidation film, potentially influencing its protective properties.

In conclusion, the testing position of consolidated soil columns significantly affects their resistance to transportation-induced vibrations. It can impact wear, stress distribution, consolidation film integrity, and the realism of the simulation. Researchers should select the testing position based on their specific research goals and the accuracy of the simulation they aim to achieve.

#### 4.2.3. The Influence of Two Different Testing Methods on the Transport Vibration Resistance Test of Consolidated Soil Columns

Compared with Test Method 1, Test Method 2 is simple and efficient. The comparison effect between different test samples is obvious, and the position of the test samples during the testing process has little impact on the test results. However, it is not very clear when studying the impact of a certain factor on test results in Method 2. While Test Method 1 may be more complex, it has a significant effect in identifying the impact of a certain test condition on the consolidated soil column. Therefore, vibration Test Method 1 and Test Method 2 have their own advantages and disadvantages and can be selected according to actual testing needs.

Regardless of which method of transportation vibration testing is used, it is has found that the factors that have the greatest impact on the wear resistance of consolidated soil columns are the viscosity, fluidity of the KGM/CA/PVA adhesive, dryness of the adhesive film, and soil particle size.

Therefore, in the practical application process of seedling transplantation, selecting the appropriate formula of KGM/CA/PVA adhesive can form a protective film on the soil ball at the root of the seedling.

## 5. Conclusions

This study employed two methods for testing the resistance to transportation-induced vibrations of two types of consolidated soil columns to assess the consolidation performance of polymer-based soil consolidation agent on the root ball of seedlings and combined the compressive test of consolidated soil columns to jointly study the factors affecting the damage resistance of soil columns/soil balls at the root of seedlings. We found that when the consolidation agent has a high viscosity and good flowability, and the soil has larger particle sizes, the resulting consolidated soil columns exhibited thicker surface films. Consequently, these soil columns show improved resistance to compression and transportation-induced vibrations. Furthermore, higher levels of drying in the consolidated soil columns lead to enhanced wear resistance. In conclusion, the adhesive film plays an important role in enhancing the transportation vibration resistance of consolidated soil columns.

Method 1 for testing the resistance of consolidated soil columns to transportation vibration is cumbersome, but the testing is more accurate, and the effect is obvious, especially when investigating the impact of a certain test or preparation condition on the consolidated soil column. Method 2 for anti-transportation vibration testing is simple and efficient, with a significant comparison of effects between samples. The position of the samples during the testing process has little impact on the test results. The study mainly provides a quick and effective method to detect the anti-damage of consolidated soil columns/balls during transportation, with an emphasis on detecting the anti-transport oscillation and anti-compressibility of consolidated soil columns/balls. As testing technology continues to advance, the detection techniques for the resistance of consolidated soil columns to transportation-induced vibrations will mature. The simulated testing results will provide increasingly valuable guidance for the practical application of soil consolidation agents in the transplantation of seedlings.

## Figures and Tables

**Table 1:** Vibration test conditions for truck transportation on expressways.

Frequency of Turning Point (Hz)	Power Spectrum Density (g^2^/Hz)	Root Mean Square Acceleration	Test Time	Test Directions
2	0.01500	1.10 g	30 min/direction	Longitudinal Direction, Transverse Direction, Vertical Direction
40	0.01500			
500	0.00015			

**Table 2:** Vibration test conditions for combined wheeled vehicle transportation.

Vibration Condition of Combined Wheeled Vehicle					
Vertical Direction		Transverse Direction		Longitudinal Direction	
Hz	g^2^/Hz	Hz	g^2^/Hz	Hz	g^2^/Hz
5	0.2366	5	0.1344	5	0.0593
8	0.6889	7	0.1075	8	0.0499
12	0.0507	8	0.1279	15	0.0255
21	0.0202	14	0.0366	16	0.0344
23	0.0301	16	0.0485	20	0.0134
24	0.0109	17	0.0326	23	0.0108
26	0.0150	19	0.0836	25	0.0148
49	0.0038	23	0.0147	37	0.0040
51	0.0054	116	0.0008	41	0.0059
61	0.0023	145	0.0013	49	0.0016
69	0.0111	164	0.0009	63	0.0011
74	0.0029	201	0.0009	69	0.0040
78	0.0048	270	0.0051	78	0.0008
84	0.0033	298	0.0021	94	0.0020
90	0.0052	364	0.0099	98	0.0013
93	0.0034	375	0.0019	101	0.0025
123	0.0083	394	0.0073	104	0.0014
160	0.0041	418	0.0027	111	0.0024
207	0.0055	500	0.0016	114	0.0014
224	0.0139	1.62 g		117	0.0020
245	0.0031			121	0.0012
276	0.0129			139	0.0024
287	0.0036			155	0.0021
353	0.0027			161	0.0034
375	0.0049			205	0.0042
500	0.0010			247	0.0303
2.20 g				257	0.0027
				293	0.0092
				330	0.0116
				353	0.0231
				379	0.0083
				427	0.0220
				500	0.0014
				2.05 g	

Test directions: vertical direction, transverse direction, and longitudinal direction; Test time: 20 min/direction.

**Table 3:** The conditions of the impact test.

Wave Form	Acceleration Peak	Pulse Width	Numbers of Impact	Test Directions
Final peak saw tooth wave	20 g	11 ms	3 times/direction	Three-axis

**Table 4:** ISTA/ASTM Standard Test Method for Simulating Transportation Vibration.

Vibration Test Sequence	Test Speed (r/min)	Corresponding Frequency (Hz)	Test Time (min)
Test 1	150	2.5	95
Test 2	180	3.0	79
Test 3	210	3.5	66
Test 4	240	4.0	60

**Table 5:** Preparation information of the sample in Figure 6.

Type of Soil Columns	Particle Size of Soil (mm)/Sample No.					
Model 1	1 Sample 1	2 Sample 2	3 Sample 3	4 Sample 4	5 Sample 5	Mixed Sample 6
Model 2	1 Sample 7	2 Sample 8	3 Sample 9	4 Sample 10	5 Sample 11	Mixed Sample 12

The preparation of polymer-based soil consolidation agent: preparation temperature 50 °C, pH of the adhesive solution 4.5, and the content of KGM, CA, and PVA is 4.5%, 4.5%, and 4.0%, respectively. The selected soil is loam in North China, with a pH of 8.5.

**Table 6:** Preparation conditions of soil consolidation agent.

Type	Preparation Conditions of the Main Agent of Consolidation Adhesive Used for Sample Treatment	Particle Size of Soil	pH of Soil
Type1	50 °C; KGM, CA, PVA (4.0%, 4.0%, 2.0%); pH 4.5	2 mm	8.5
Type 2	60 °C; KGM, CA, PVA (4.5%, 4. 5%, 3.0%); pH 4.5	2 mm	8.5
Type 3	65 °C; KGM, CA, PVA (5.0%, 5.0%, 5.0%); pH 4.5	1–5 mm	8.5

**Table 7:** Sample details of consolidated soil columns.

Sample Number	Preparation Conditions of the Main Agent of Consolidation Glue Used for Sample Treatment	Particle Size of Soil	pH of Soil
Sample 1	Blank Control, Not Consolidated with Adhesive	3 mm	8.5
Sample 2	30 °C; KGM, CA, PVA(4.5%, 4.5%, 3.0%); pH 4.5	3 mm	8.5
Sample 3	40 °C; KGM, CA, PVA(4.5%, 4.5%, 3.0%); pH 4.5	3 mm	8.5
Sample 4	50 °C; KGM, CA, PVA(4.5%, 4. 5%, 3.0%); pH 4.5	3 mm	8.5

## Data Availability

This will be made available upon request through the corresponding author.
